# Business climate and firm exit in developing countries: evidence from Nigeria

**DOI:** 10.1186/s43093-022-00160-6

**Published:** 2022-11-05

**Authors:** Ebuka Christian Orjiakor

**Affiliations:** Department of Economics, University of Nigeria, Nsukka, Nigeria

**Keywords:** Business climate, Firm-level constraints, Firm exit, Private sector, Developing economies, D22, D81, L25

## Abstract

The business environment goes a long way to influence firm performance through several channels such as government policies and regulations, institutions, infrastructure, and macroeconomic variations. With developing countries known for having the most deteriorating business climate, this study set out to answer the question: "Does business climate account for firm exit in developing countries?" Using a comprehensive dataset of panel firms from the World Bank Enterprise Survey in Nigeria, the study aggregates 15 firm-level constraint variables into a composite index and estimates the likelihood of firm exit using the binary probit model. The results from the analysis infer that the unconducive state of the business climate in Nigeria significantly impedes firms’ prospects for survival. More specifically, the estimated probability of exiting the market is predicted to grow by 11% points for every additional increase in the constraint index. The recommendation follows that efforts should be geared toward improving the state of the business climate in Nigeria through carefully designed policies that can foster private sector development. Such policies should among others increase government investment in critical infrastructure, eliminate destructive tax policies, and maintain a healthy macroeconomic environment, which by implication, go a long way to improve business longevity and contributes to national development.

## Introduction

The private sector remains the main engine of development for every economy and most importantly for developing countries where it absorbs the unemployed human capital and plays a meaningful role in reducing global poverty. This explains the efforts, energy, and resources that are being put in place by researchers, think tanks, and development institutions to promote the sector’s development through various projects and programs. An example is the recent Business Enabling Environment (BEE) Project by the World Bank Group that provides a new methodology for assessing the global business environment with the goal to foster development in the private sector [1].

It is a well-known fact that the private sector’s growth is driven by the innovation and entrepreneurship behavior of individual entrepreneurs. Notwithstanding, the business environment through several channels (such as government regulations and policies, institutional quality, variations in the macroeconomic environment, infrastructure availability, market structure or composition, etc.) goes a long way to influence the sector’s development and developing countries known to have a worst deteriorating business climate that impedes business operations [2, 3].

Nigeria, despite being the largest economy in Africa is faced with diverse challenges in her business environment, ranked 131st out of 190 nations by the World Bank Ease of Doing Business in 2020 [4]. Numerous challenges confront the country’s business environment with insecurity as the most pressing obstacle. The North East part of the country has witnessed a decade of terrorist attacks by bandits and militant Islamist Boko Haram groups, with spreading violence and insecurity in the North West and Middle Belt [5]. Similar to that, the Niger Delta region that houses the nation’s oil wells is faced with conflict attacks from the Niger Delta militants, coupled with other crimes like kidnapping, police brutality, and farmers/herdsmen conflict that are prevalent in other parts of the country. The country’s public institutions are not without their challenges; characterized by high levels of inefficiency, corruption, and lack of technological dynamism. Among many others, inefficient infrastructure has over the years remained the major challenge to the country’s prospect for development [6]. The country’s infrastructure is ranked 130th out of 138 countries in the 2019 Global Competitiveness Index, with utility and transport infrastructure ranking 124th and 130th, respectively [7]. In this vein, the World Bank enterprise survey noted insufficient infrastructure as one of the major hindrances to business operations in Nigeria [8].

**Fig. 1:**
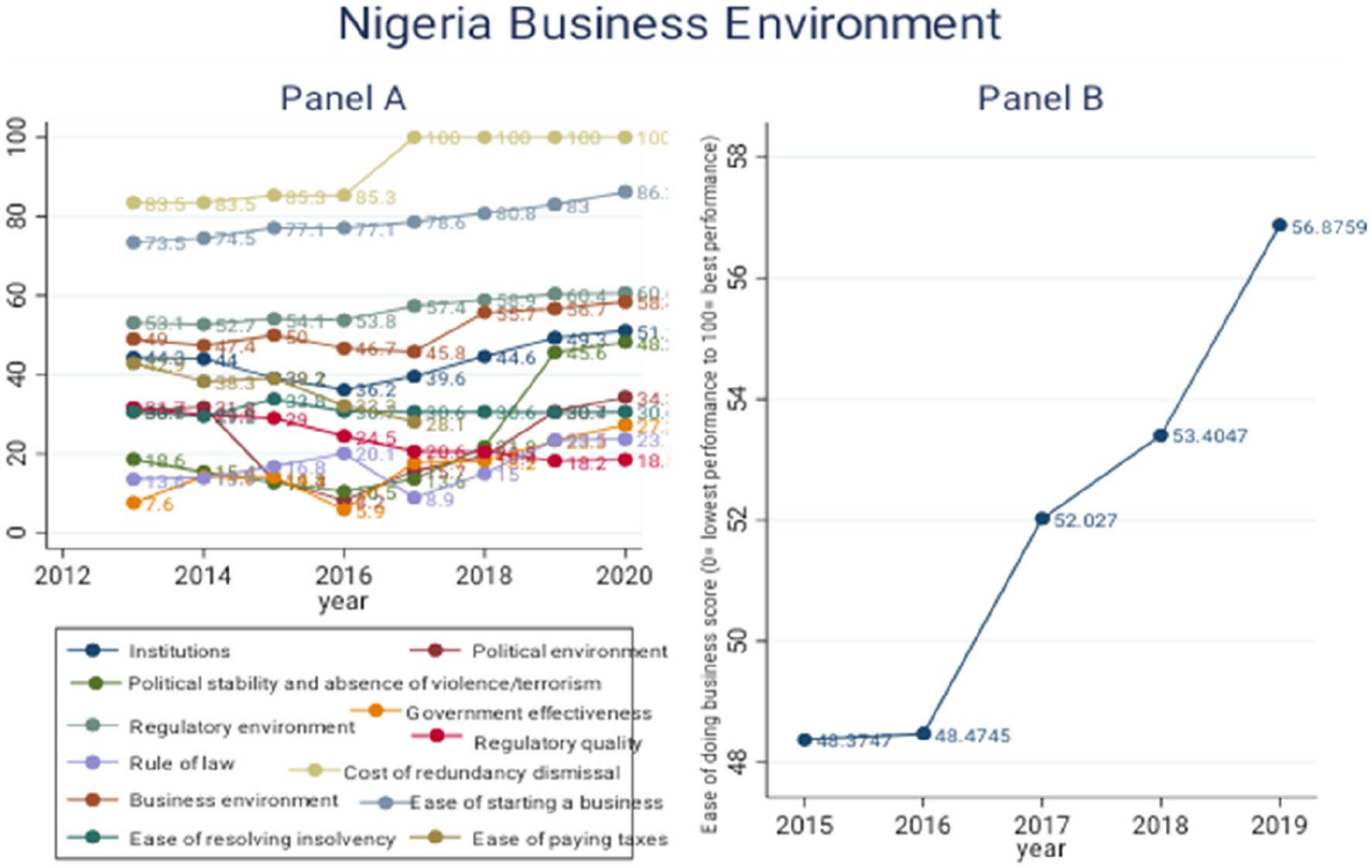
Nigeria’s business environment. Source: Author’s Computation using Data from Global Innovation Index and World Development Indicator

Figure 1 displays a snapshot of Nigeria’s business and investment climate using data from the Global Innovation Index and World Bank’s World Development Indicators. The first part of the diagram (panel A) shows the score of selected variables from the Global Innovation Index. Except for the *cost of redundancy dismissal*, higher values of the indices indicate better outcomes.

The first panel of Fig. 1 shows that the *ease of starting a business* in Nigeria has improved in recent years, with a score value that is above 70. In most recent times, between 2016 and 2020, indicators like *rule of law, regulatory environment,* and *government effectiveness* have their score values below 30, while the *cost of redundancy dismissal* has also increased in recent times. This implies that the country’s institutional environment is marked by some level of weakness in recent times. The second panel indicates a gradual improvement in the *ease of doing business* in Nigeria.

**Fig. 2:**
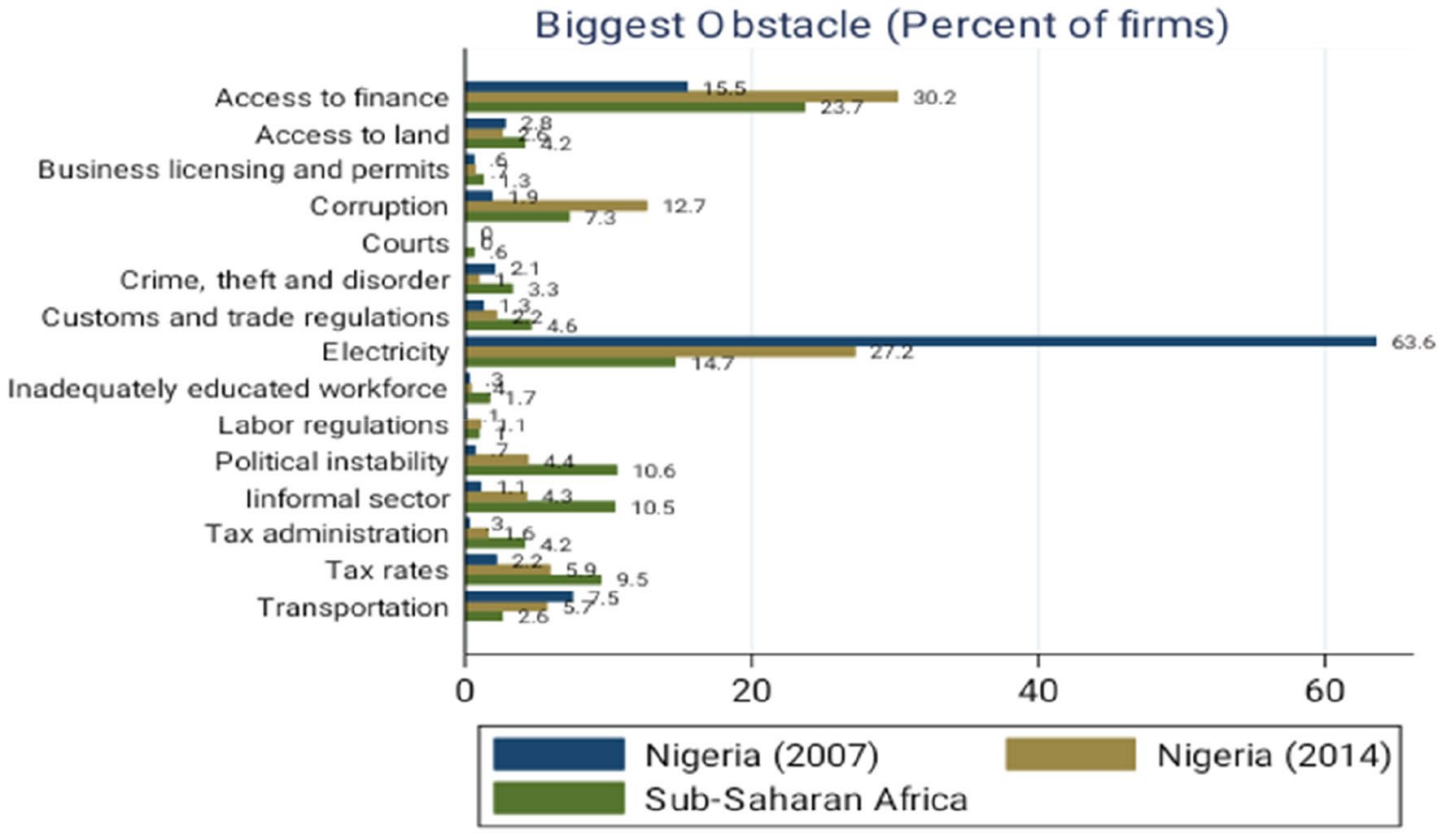
Biggest obstacles to firms’ operation in Nigeria. Source: Author’s Computation using Enterprise Survey Data

There is no doubt that the deplorable state of the business climate in Nigeria has hampered firms’ activities in several ways. Among earlier studies on Nigeria’s business environment, Adenikinju [9] argued that the poor power supply in Nigeria has made many start-ups enterprises spend equivalently 20–30% of their initial investment on costly self-generation in their bid to mitigate the cost of frequent outages. Similarly, Okechukwu [10] provided evidence for the cost of power failures on manufacturing firms’ capacity utilization in Nigeria. The World Bank Enterprise Survey likewise identified obvious factors that hamper firms’ performance in Nigeria such as access to finance, corruption, stifling business regulations, crime, an inadequate workforce, political instability, informal competition, and inadequate infrastructure [8]. Figure 2 shows the percentage of Nigerian firms in two rounds of the enterprise surveys (2007 and 2014) that identify these factors as the biggest obstacles to their operation in comparison with other Sub-Saharan African countries.

Compared to other Sub-Sahara African countries, Nigeria has the least percentage of firms identifying *Court; Crime, Theft and Disorder; Custom and Trade Regulation; Inadequate Educated Workforce; Political Instability; Informal Sector; Tax Administration;* and *Tax Rate* as the biggest challenges to their operation. However, worrisome challenges exist in other areas and loom the largest in *Access to Finance* and *Electricity.* About 30.2% of the surveyed firms in 2014 identified *Access to Finance* as the biggest constraint to their operation against 15.5% in 2007. Likewise, 63.6% of the surveyed firms in 2007 identified electricity as the biggest obstacle to their operation, a value that is many times higher than that of other African nations. In 2014, the percentage of firms identifying electricity as the biggest obstacle reduced to 27.2% which signals an improvement in the country’s electricity sector.

Several existing studies have assessed the effect of different dimensions of business climate on firms’ performance and growth. An increasing number of them examined the effect of infrastructure on firms’ performance [2, 11–16], others examined the importance of access to finance [3, 17–21], institutions and regulatory environment [22–24], and the cost of bribery and corruption [25–27].

Generally, these studies conclude that an unfriendly business environment harms firm productivity, raises costs and risks of doing business, and creates barriers to competition. However, studies that examine the importance of business climate in determining firm survival/exit remain sparse in literature (with exception of [28–31]. Even though the possibility abounds that an unfriendly business climate can impede survival and/or increase exit, most business survival literature focused on the internal factors that explain firm survival (e.g. [32–35]).

This study aims to contribute to the knowledge gap by investigating how business climate account for firm exit in Nigeria using firm-level data from the World Bank Enterprise Survey. Evidence from the study maintains that an unfriendly business climate adversely affects firms’ success in business, thereby making them more susceptible to exit. The remainder of this paper is arranged as follows: Sect. 2 reviews the related literature, Sect. 3 gives attention to the methodology and data source used in the study, Sect. 4 discusses the analytical results, and Sect. 5 provides the conclusion.

## Literature review

A review of relevant business theories suggests that firm success is determined by internal or/and external factors in the business environment, while the harsh forces of competition ensure that only successful firms remain in business and the unsuccessful ones exit. The supporters of the resource-based view uphold that a firm source of competitive advantage depends on the number of resources and competencies internal to the firm. They contend that firms will have varied types of resources and levels of capability and firms that efficiently use such resources and skills will outperform others [36]. In this vein, the proponents of the competence-based view contend that firm resource endowment is insufficient to explain performance variations among firms, instead, firm success should be a question of how effective and/or efficient it uses its available resources [37]. On the other hand, the Jovanovic [38] theory of noisy selection claims that a firm's survival is determined by the level of firm adjustment efficiency over time. As a result, according to the hypothesis, efficient firms develop and flourish whereas inefficient firms deteriorate and collapse. According to the survival-based hypothesis, the condition for survival is based on the willingness to implement strategies that focus on maintaining extremely efficient operations and being able to adjust quickly to changing competitive environments [39]. Also, the Schumpeterian creative destruction theory supports the notion of efficiency based on innovation being the precursor to whether a firm survives competition and expands or dies [40].

Several existing business climate literature provided evidence for the effect of business obstacles such as infrastructure, finance, institutions, corruption, and business regulation on measures of firm performance and growth. Talking about infrastructure such as electricity, several studies argued that adequate and reliable electricity matters for firm performance. In this regard, Fakih et al. [13] used measures of firm performance such as sales, employment, and productivity growth rates to examine the impact of power outages on manufacturing firms’ performance in the Middle East and North Africa region using data from the World Bank enterprise survey. The study provided evidence that power outages in all their forms have adverse effects on manufacturing firm performance and are much evident in sales and labor productivity growth rates. Similarly, Iimi [14] established that frequent electrical outages increase corporate costs for enterprises in 26 transition economies in Eastern Europe and Central Asia, while Abdisa [11] observed that investing in self-generation reduces outage loss for firms that invest in it, although such firms continue to experience greater unmitigated outage loss than firms that did not invest in self-generation. On the other hand, Geginat and Ramalho [2] identified the level of bureaucracy in low-income countries to be a significant factor that explains the inefficiency in utility distribution in these countries and supports the premise that electricity connectivity matters for firm performance. The study indicated that firms that face smaller and less costly electricity connection processes have better performance and most especially in sectors with high electricity needs.

Related studies examined the general impact of infrastructure quality on business performance. Findings from Escribano et al. [12] for manufacturing enterprises in 26 African countries established that infrastructure quality has a high negative impact on low-income countries’ total factor productivity and a minor positive influence on high-income countries’ total factor productivity, while Iimi et al. [15] revealed that infrastructure quality is essential for enhancing enterprise productivity in selected five East African countries. Rentschler et al. [16], on the other hand, estimated the monetary cost of unreliable infrastructure for firms in 137 low- and middle-income nations to be around $300 billion each year, with annual utilization losses of $151 billion.

Another body of literature channeled concern to the institutional aspect of the business environment, specifically governance, regulation, and corruption and investigate their impact on firm growth and performance, and access to credit and public utilities. For example, Yang [24] used firm-level data for small and medium enterprises in Latin America and the Caribbean region to provide evidence that a weak environment reduces the performance of innovative SMEs compared to their counterpart. In the same vein, Amin and Ulku [25] used firm data for more than 39,000 enterprises in 111 countries to show that corruption adversely affects firm productivity and is highly significant in times of high regulation. Ayyagari et al. [22] provided evidence that innovative firms especially those in countries with excessive bureaucratic regulations and weak governance institutions are more likely to face rent-seeking from government officials and still do not face better service delivery. In this vein, Freund et al. [27] showed that contrarily to popular opinion that demand for bribes is associated with quick service delivery, the amount of time to secure an operating license, building permit, or electrical connection is 1.5 times longer for firms that are faced with bribes demands than their counterpart, 1.2 times longer to clear customs when exporting, and 1.4 times longer when importing. Amin and Motta [26] also established evidence that corruption in developing countries strongly limits SMEs' access to credit and estimated that with every increase in bureaucratic corruption, say from the minimum to maximum value, SMEs' chances of being financially constrained increase from 6.9 to 10.9% points.

In addition to some of the mentioned constraints, factors such as lack of access to finance, competition from the informal sector, and crime are also recognized in business literature as key constraints to business success. Essmui et al. [21] applied the structural equation model (SEM) to analyze the impact of a deplorable business environment on manufacturing firms’ employment growth using enterprise data from 207 enterprises operating in three commercial cities in Libya. The study identified lack of finance, crime, human capital, corruption, and infrastructure to be significant factors that obstruct the employment growth of firms in Libya but failed to obtain evidence for the effect of competition and business regulations on firms’ employment growth. Similarly, Aterido et al. [18] used enterprise data for more than 56,000 establishments in developing and high-income economies to study the effects of the business environment, mainly infrastructure, access to credit, corruption, and business regulations on firms’ employment growth while accounting for heterogeneity across firm size. Evidence from the study indicated that the employment growth rate for medium and large firms is adversely affected by lack of access to credit and deplorable infrastructure, while that of small firms is mostly affected by business regulations.

In a similar study, Klapper et al. [23] obtained evidence that formal sector growth measured by firm entry and density rates are robustly related to measures of a country’s economic growth and development, level of legal and institutional (regulatory) development, ease of access to finance, and activities of the informal sector. Further evidence from the study supported the premise that business environment proxy by the ease of starting a business and political corruption are significant predictors of the number of firm registrations. Applying methods of regression analyses and directed acyclic graph methodology, Ayyagari et al. [19] revealed that business constraints on finance, political instability, and crime are binding constraints that have a direct influence on firm growth, with finance having the largest impact. Ullah [41] also indicated that inadequate finance hurts the sales and employment growth of SMEs in 28 Eastern European and Central Asian countries after accounting for differences in countries' levels of development, institutional quality, and corruption. Amin and Viganola [17] obtained similar evidence that firms with access to finance before the Covid-19 pandemic have a lower likelihood of experiencing decreased sales during the pandemic, while Bahy and Cooper [20] identified lack of access to credit and degree of competition as major constraints limiting income growth of small firms in Northern Myanmar.

Aterido et al. [42] examined the impact of credit access, business regulation, corruption, and infrastructure on the employment growth of 70,000 firms in 107 countries and found evidence of composition effects of business environment on firm employment growth, suggesting that weak business climate adversely affect employment growth of firms. The effects of access to finance and regulation reduce the employment growth of all enterprises, most especially, micro- and small enterprises, while corruption and deplorable infrastructure reduce the employment growth of medium and large enterprises. In a comparison study of Africa and the rest of the world, Aterido and Hallward-Driemeier [3] assessed how access to credit, infrastructure, regulatory environment, and corruption affect patterns of employment growth in Sub-Sahara Africa using World Bank Enterprise Survey for 104 countries including 31Sub-Sahara African countries. The authors argued that even though Sub-Saharan Africa has a more challenging investment climate than the rest of the world, it does not translate to low employment growth. Instead, more of the major constraints; particularly, access to finance and infrastructure translate to expanding micro-enterprises. The effect of power outages is found to lower the employment growth of large firms in the region but promotes the employment growth of micro firms.

Another body of literature took a different approach to examine the impact of the business environment on firm entry and choice of entry. For example, Klapper et al. [43] used firm-level data for Western and Eastern Europe to establish evidence that entry regulations hamper the rate of firm entry and are more evident in sectors that naturally should have a high rate of entry, which further translates to lower output per worker in the sector, especially for countries with burdensome regulations on entry. In this manner, Klapper et al. [44] used data from business registries to provide evidence that better governance and lesser burden in starting a business such as a fast, efficient, and cost-effective business registration process are important factors that drive entrepreneurship activity in the formal sector. Also, Klapper and Love [45] used longitudinal data on new firm registrations in 91 countries to show that the number of new firm registrations is significantly determined by the required costs, days, and procedures of starting a business.

As pointed out in the introduction, most survival literature focused on the internal antecedents of firm survival/exit such as firm age, size, innovation capabilities, and ownership structure. In this regard, Esteve-Pe´rez and Man˜ez-Castillejo [34], Dunne and Masenyetse [33], Aga and Francis [32], and Esteve-Pérez and Sahiti [35] found the effect of business size and age to robustly increase firm survival. Also, Varum and Rocha [46] provided evidence for the moderating effect of firm size on firm exit during economic downturns. These studies agreed that small firms have more exit likelihood compared to large firms. Using data from new firms created in the Netherlands in 2001–2006, Cefis and Marsili [47] investigated the association between firms' innovative capabilities and survival during and after the 2007–2008 global financial crises. The study found evidence that new firms that venture into innovation within two years of operation benefited from lasting adaptive survival premiums during and after the crisis.

Most empirical studies on business climate and firm survival/exit are outside the context of developing countries. For example, Hallward-Driemeier [48] used a dataset of enterprises operating in 27 Eastern European and Central Asian countries to establish that inefficiencies in the business environment, specifically, access to credit, the efficiency of public services, corruption, level of competition, and strength of property rights are associated with a higher risk of business exit. Similarly, Iwasaki et al. [28] supported the premise that quality institutions and developed financial systems help improve firm longevity, using a dataset of 94,401 small enterprises in 17 European emerging markets from 2007 to 2017. In the same manner, Klapper and Richmond [29] used data from registered businesses in Cote d’Ivoire for the period 1976–1997 to show that the risk of firm exit increases with types of reforms, while the firm likelihood of survival increases monotonically with firm size and better economic performance, while Muzi et al. [30] established that excessive regulation proxy by the amount of time senior executive spent dealing regulatory requirements increases the risk of firm exit during the Covid-19 pandemic. Orjiakor and Omeje [31] obtained evidence that firms with improved access to infrastructures such as electricity, telecommunication, transportation, and quality institutional services have better chances of survival in Nigeria’s business environment than those without such access.

From the review, it is evident that there is a literature gap to be filled in the area of business climate and firm survival/exit, especially in the context of developing countries and Nigeria precisely. In other to bridge this gap and contribute to knowledge, this study provides fresh insights into how business climate accounts for firm exit in Nigeria.

## Methodology

### Data source

This study utilizes the harmonized Enterprise Survey data for Nigeria in the period 2007–2014. The World Bank Enterprise Surveys (ES) provide firm-level data on business and investment climate in 151 countries. The surveys are representative samples of an economy’s private sector and contained a broad array of indicators that are used to study the business environments such as infrastructure, corruption, finance, competition, crime, and performance indicators. The availability and the unique qualities of the survey make it the most suited dataset for this study. To the best of the author’s knowledge, the Nigeria Enterprise Survey is the only available dataset that provides firm-level data of a representative sample of Nigeria’s private sector comprising both the manufacturing and service sector. In addition, the dataset identifies obvious factors that hamper firm performance such as access to finance, corruption, stifling business regulations, crime, inadequate workforce, political instability, informal competition, inadequate infrastructure, and also firm demographic information. This information helps to discern constraining factors that affect business survival. Finally, the harmonized dataset contains information on the operating state of all previously surveyed enterprises including enterprises that have exited the market. This information enables researchers to follow up over time on changes in the business environment and as well, investigate the dynamics of firm survival/exit between two survey rounds [32].

### Measurement of variables

#### Business climate

The measure of business climate used in this study is based on firms’ self-reported perceptions of the business environment. In addition to objective measures of the business environment, the enterprise survey includes individual firms’ assertions, on a scale of 0 (no obstacle) to 4 (very severe obstacle), about a number of external factors in the business environment that influence business activities. It can be argued that such subjective measures can be potentially flawed as they are based on firms’ perceptions rather than their direct experiences in the environment. However, a potential problem with most objective indicators in the ES dataset is that they are likely to have missing data for a number of the observations, most especially when the concerned variable is peculiar to some firms in the sample. For instance, questions like “number of days to clear custom” is distinct to only importing/exporting firms in the sample.

In an attempt to have a holistic view of the business climate in Nigeria, the study identifies 15 subjective indicators that assess the business environment and recode them into binary variables: equals 1 if a firm reported the concerned item/factor as “major obstacle” or “very severe obstacle” to its current operation, and 0 if otherwise (see Table 1). Indicators such as electricity, access to finance, and tax rate have the highest number of firms identifying them as a major or very severe obstacle to their operation. About 84% of firms in the sample reported electricity as a major or very severe constraint to their operation, while 51% of them identified access to credit as a major or very severe obstacle to their current operation. On the other hand, just a few of the firms in the sample (less than 8%) considered factors like telecommunication, custom and trade regulation, and labor regulation as a significant obstacles to their operation. By the way of average, the author aggregates these indicators into a composite index (referred to as the business constraint index). The index is constructed such that larger values indicate higher levels of constraints in the business environment and are bounded between 0 (zero constraints) and 1 (full constraints). The item-test correlation shows that the individual indicators are positively correlated with the constraint index and have a coefficient value between 0.2460 and 0.5522. The Cronbach’s alpha coefficient of *α* = 0.6213 further indicates that the index has good internal reliability. To test the consistency of the index as a measure of business condition, a simple correlation analysis is performed between the constraint index and some quantitative indicators in the ES dataset. The result indicates a positive and strong correlation between the constraint index and a good number of variables used in the analysis.^1^

**Table 1:** Indicators used in generating the business constraints index. *N* = 1157

Indicator code/unit	No. of firms (%)	Item-test correlation
*Electricity:* takes the value of 1 if electricity is a severe or extremely severe obstacle to the firm's existing activities, and 0 if it is a moderate, small, or nonexistent difficulty	83.66	0.2460
*Telecommunication:* takes the value of 1 if telecommunication is a severe or extremely severe obstacle to the firm's existing activities, and 0 if it is a moderate, small, or nonexistent difficulty	7.87	0.3096
*Transportation: *takes the value of 1 if transportation is a severe or extremely severe obstacle to the firm's existing activities, and 0 if it is a moderate, small, or nonexistent difficulty	31.89	0.4327
*Custom and trade regulation:* takes the value of 1 if custom and trade regulation is a severe or extremely severe obstacle to the firm's existing activities, and 0 if it is a moderate, small, or non-existent difficulty	5.013	0.2481
*Tax administration:* takes the value of 1 if tax administration is a severe or extremely severe obstacle to the firm's existing activities, and 0 if it is a moderate, small, or nonexistent difficulty	28.09	0.5522
*Tax rate:* takes the value of 1 if the tax rate is a severe or extremely severe obstacle to the firm's existing activities, and 0 if it is a moderate, small, or nonexistent difficulty	35.35	0.4925
*Business licensing:* takes the value of 1 if the tax rate is a severe or extremely severe obstacle to the firm's existing activities, and 0 if it is a moderate, small, or nonexistent difficulty	17.72	0.4296
*Political instability:* takes the value of 1 if political instability is a severe or extremely severe obstacle to the firm's existing activities, and 0 if it is a moderate, small, or nonexistent difficulty	16.68	0.4016
*Corruption:* takes the value of 1 if corruption is a severe or extremely severe obstacle to the firm's existing activities, and 0 if it is a moderate, small, or nonexistent difficulty	32.50	0.4976
*Crime, theft, and disorder:* take the value of 1 if crime, theft, or disorder is a severe or extremely severe obstacle to the firm's existing activities, and 0 if it is a moderate, small or nonexistent difficulty	23.51	0.4406
*Courts:* takes the value of 1 if the court system is a severe or extremely severe obstacle to the firm's existing activities, and 0 if it is a moderate, small, or nonexistent difficulty	10.46	0.3063
*Access to land:* takes the value of 1 if access to land is a severe or extremely severe obstacle to the firm's existing activities, and 0 if it is a moderate, small, or nonexistent difficulty	23.34	0.3973
*Labor regulation:* takes the value of 1 if labor regulation is a severe or extremely severe obstacle to the firm's existing activities, and 0 if it is a moderate, small, or nonexistent difficulty	7.78	0.3481
*Competitors in the informal sector: *takes the value of 1 if the activity of informal competitors is a severe or extremely severe obstacle to the firm's existing activities, and 0 if it is a moderate, small, or nonexistent difficulty	17.46	0.3670
*Access to finance:* takes the value of 1 if access to finance is a severe or extremely severe obstacle to the firm's existing activities, and 0 if it is a moderate, small, or nonexistent difficulty	51.08	0.4232
Test scale:		0.6213

### Firm exit

As stated earlier, information in the ES dataset is useful for investigating firms’ survival/exit dynamics in the elapsed period between two survey rounds. This information is used to construct a binary indicator of firm exit that takes the value of 1 if a firm is considered to have exited the market, and 0 if otherwise. In classifying “exited” firms, this study follows Aga and Francis [32] conservative definition of firm exit which is based on the idea that firms in business would remain in business until it is proven beyond every reasonable doubt that they have exited the market. Following the classification of “weak exit” in Aga and Francis [32], a firm is considered to have exited the market if: (1) it is established during the screening process that the firm is out of operation, (2) firm activities now relate to an ineligible activity or status, such as moving abroad, becoming fully owned by the government, engaging in out-of-universe activities, or has withdrawn its membership with a trade association, or (3) all additional attempts to gather contact information prove abortive after the listed contact information turns to be a deadline or a non-functioning phone line, and lastly, (4) contact information is misleading and there are no accessible new records [32].

### Model specifications for exit predication

Following Hallward-Driemeier [48] and Aga and Francis [32], this study adopts a binary outcome model to examine the effect of business environment constraints on firms’ likelihood of exiting the market. The exit model is represented with the help of a binary choice equation below:

1 $${\text{exit }} = \{ 1 \;{\text{if}}\; Q_{t} < Q^{*} ;\; \;0\; {\text{otherwise}}$$

Equation (1) represents the firm’s choice of exit, where $${Q}_{t} and {Q}^{*}$$ denote the firm’s current performance level and optimal performance level, respectively. The exit model is built on the assumption of perfect competition, such that an enterprise operating in an environment full of risks will choose or be compelled to quit the market if its current level of productivity ($${Q}_{t})$$ falls behind certain optimal point $${(Q}^{*})$$ [32]. The model is conceptualized using the method of latent variable in Eq. (2) and estimated by the binary probit regression approach.

2 $$y_{i}^{*} = X_{i}^{\prime } \gamma + \mu_{i}$$

The latent or unobserved variable $${{(y}_{i}}^{*})$$ represents $$ith$$ firm’s risk of exit and is influenced by a vector of observed explanatory variables ($$X_{i}^{\prime } )$$ which includes the business constraints index and a host of firm-level controls; $$\upgamma$$ is the associated coefficients vector; and $${\mu }_{i}$$ is the error term. The error term in a probit model follows the assumptions of normal distribution, with zero mean [*E*($$\mu$$) = 0] and constant variance [Var($$\mu$$) = *σ*] [49]. Using a simple measurement equation (Eq. 3) further simplifies the relationship between the latent and observed binary outcome ($$y_{i} )$$.

3 $$y_{i} = \left\{ {\begin{array}{*{20}c} 1 & {{\text{if}}\; y_{i}^{*} > 0} \\ 0 & {{\text{if }}\;y_{i}^{*} \le 0} \\ \end{array} } \right.$$

Here, it is assumed that a firm choice or risk of exit is determined by a latent factor (say the propensity to remain in business) that cannot be directly measured. Firm exit is observed ($${y}_{i}=1)$$ when the latent factor is positive ($${{y}_{i}}^{*} >0$$) and unobserved ($${y}_{i}=0)$$ if otherwise ($${{y}_{i}}^{*} \le 0)$$. The functional form of the latent variable model for binary outcomes can be expressed as:

4 $$\Pr \left( {y = 1|X^{\prime}} \right) = \Pr \left( {y^{*} > 0|X^{\prime}} \right)$$

The functional form of the exit model is expressed as:

5 $$\Pr \left( { {\text{exit}}_{t + n} } \right) = \Pr \left( {y = 1} \right) = {\text{BusCons}}_{{{\text{it}}}} , {\text{FirmCont}}_{{{\text{it}}}} , {\text{FE}}_{{{\text{it}}}}$$

where $Pr({ exit}_{t+n})$ is the probability that $ith$ firm will exit the market at time $t+n$. $BusCons$ denotes the business constraints index; $FirmCont$ represents firm-level controls included in the model; and $FE$ corresponds to industry, location, and year control dummies (fixed effects). As often pointed out in the micro-econometric literature (e.g., [12], regressions on a limited number of explanatory variables are likely going to produce biased results arising from omitted variables. A recommended approach in the literature is to include control variables in the model.

The first set of controls in the model relates to firm demographics such as the firm’s age and size, usually referred to as the “liability of newness” and “liability of smallness.” Empirical evidence suggests that small and young firms have a higher risk of exit than old and large firms. For example, Esteve-Pe´rez and Man˜ez-Castillejo [34]; Dunne and Masenyetse [33], Aga and Francis [32], and Esteve-Pérez and Sahiti [35] found the effect of business size and age to robustly increase firm survival. A popular explanation in the literature is that larger firms have production levels close to the minimum efficient scale (MES) and therefore are less vulnerable than small firms that operate at a lower scale. Also, larger enterprises are known to be more diversified than smaller enterprises, thus their risk of withdrawal or termination because of poor market conditions could be compensated by stronger market conditions elsewhere.

The ownership structure of the firm is another factor in the literature that influences firm survival prospects. This relates to whether a firm has the presence of foreign ownership and/or the presence of female top owners or managers. A common belief is that firms with international status are more likely to survive because they tend to benefit from local policies designed to encourage foreign investment and at the same time have better access to advanced technology and financial resources. However, the possibility exists that foreign ownership can increase exit likelihood in scenarios where foreign-owned firms are less adaptive to the local business environment or such that the business climate discourages foreign participation. For example, Esteve-Pe´rez and Man˜ez-Castillejo [34] observed the absence of foreign capital participation to increase firm survival. In the same vein, empirical evidence suggests that the gender of the firm owner also matters for firm survival (e.g., [50]). The role of performance in explaining firm survival dynamics remains inconclusive in the literature. Studies like Salmon et al. [51], Aga and Francis [32], and Muzi et al. [30] provided evidence for the improving effect of performance on firm survival, while Bosio et al. [52] observed that firm survival times are not linked to higher productivity. Additionally, this study controls for other firm-level factors that are identified in the literature such as the firm’s export status [34], investment in fixed assets, and manager’s year of experience [31].

The next set of covariates relates to industry, location (region), and year controls which account for unobservable factors that can influence firm exit. The industry dummies control for industry-specific factors such as the level of competition and/or innovation that explain firm exit. For example, firms in highly competitive industries are expected to face a higher degree of exit risk compared to firms in less competitive sectors. Similarly, the location and year dummies control the effect of firm location and time of the survey, respectively. Table 2 describes the variables used in the analyses.

**Table 2:** Variable description

Variable name	Description
*Firm exit*	Dummy variable equals 1 if the firm is considered to have exited the market at the time of the second survey round (2014); 0 otherwise
*BCIndex*	Business constraint index; a unit increase in the variable implies additional constraints to firm operations
*Age (log)*	Log of firm age; firm age equals the year of the first round survey minus the year the firm began operations
*Size (log)*	Log of firm size; firm size is the total number of the firm’s permanent employees at the end of the previous fiscal year
*Managers exp (log)*	Log of the number of years the firm's senior executive has worked in the industry
*Export*	Dummy variable equals 1 if the firm made non-national sales in the previous fiscal year; 0 otherwise
*Foreign owners*	Dummy variable equals 1 if the firm has a presence of foreign capital participation; 0 otherwise
*Female owners*	Dummy variable equals 1 if females are among the owners of the firm, 0 otherwise
*Labour prod (log)*	Log of labor productivity; labor productivity is the ratio of total annual sales to the total permanent workforce
*Fixed asset*	Dummy variable equals 1 if the firm borrowed from the bank to purchase fixed assets in the previous year, 0 otherwise
*MSMEs*	Dummy variable equals 1 if the business is a micro, small or medium enterprise, 0 otherwise
*Industry*	Industry dummies
*Region*	Regional dummies
*Year*	Year dummies

## Results and discussion

### Descriptive statistics

Table 3 shows the summary statistics for a sample of 1,157 panel firms used in the analysis. According to the definition of firm exit provided in the preceding section, 33% of the firms in the sample are considered to have exited the market by the second round of the survey. The average score of the business constraint index is estimated at 0.262. Multiplying the value by the number of items used in constructing the index indicate that, on average, firms in the sample identified at least 4 items as major or significant obstacles to their current performance. On average, firms in the sample have existed for 16 years consist of 15 permanent workers and have past annual sales per worker ratio of 751,157 naira (in nominal value), while business managers in the sample have an average experience of 8 years working in the industry. Little few firms in the sample (less than 3%) made abroad sales in the previous year and have the presence of foreign capital ownership (less than 2%). The majority of firms in the sample (85%) have at least a woman as part of firm owners and over 96% belong to micro-, small, and medium enterprises (MSMEs).

**Table 3:** Descriptive statistics of the variables

	Obs	Mean	Exp(mean)	Std. Dev	Min	Max
*Firm exit*	1,157	.3292999	n.a	.4701622	0	1
*BCIndex*	1,157	.2615961	n.a	.1569523	0	.8666667
*Age (log)*	1,157	2.76747	15.918	.4070782	1.792	4.205
*Size (log)*	1,157	2.695379	14.811	.8990181	1.609	6.551
*Managers Exp (log)*	1,157	2.125171	8.3743	.6779391	0	3.9120
*Export*	1,157	.0293863	n.a	.1689599	0	1
*Foreign Owners*	1,157	.011236	n.a	.1054482	0	1
*Female Owners*	1,157	.8530683	n.a	.3541909	0	1
*Labour Prod (log)*	1,157	13.52937	751,157	1.112175	10.637	18.603
*Fixed Asset*	1,157	.0777874	n.a	.2679525	0	1
*MSMEs*	1,157	.9619706	n.a	.1913499	0	1

### Empirical findings

Table 4 presents the estimation results. It begins by specifying a parsimonious regression model with the business constraints index as the only explanatory variable, controlling only for industry and regional effects. For inclusive readership, the coefficients of industry and regional dummies and the constant are not reported. At the same time, the year fixed-effect is omitted in the estimations due to the high collinearity. The estimated coefficients from the probit regression are reported in the average marginal effects and are interpreted based on the direction of impact and statistical significance (at conventional 10%, 5%, and 1% levels of significance). Robust standard errors clustered by firm sampling size are used in the regressions to account for clustered data and the possibility of residual correlation across time for a given firm [49]

**Table 4:** Probit regression estimates: average marginal effects. Dependent Variable: *Firm Exit*

	(1)	(2)
*BCIndex*	0.0956** (2.41)	0.105*** (3.13)
*Age (log)*		− 0.0123 ( − 0.58)
*Size (log)*		− 0.0565*** ( − 4.01)
*Managers Exp. (log)*		0.0302 (1.15)
*Export*		− 0.105 ( − 1.39)
*Foreign Owners*		0.0662 (0.46)
*Female Owners*		− 0.0382*** ( − 2.66)
*Labour Prod (log)*		− 0.0107 ( − 1.21)
*Fixed Asset*		− 0.118** ( − 2.19)
*MSMEs*		0.0816** (2.16)
*Industry*	Yes	Yes
*Region*	Yes	Yes
*Year*	No	No
*N*	1157	1157

*t* statistics in parentheses *p < 0.10, **p < 0.05, ***p < 0.01

Interestingly, the results suggest that the adverse condition of the business climate in Nigeria significantly increases firm exit from the market as indicated by a positive and significant association between measures of the business condition and the exit variable. On average, the probability of exiting the market is predicted to grow by 11 percentage points for every additional increase in the business constraint index. The finding holds (i.e., remains positive and significant) in both estimations: with or without controlling for firm demographics.

Regarding the control variables, findings from the analysis support the premise in the literature that a firm risk of exit decreases with size. The probability of firm exit is estimated to decline by 6 percentage points for a 1% increase in firm size. However, there is no significant evidence that older firms have a higher likelihood of survival nor do managers’ years of experience matter for firm survival. The finding for the effect of firm age can be attributed to the simultaneity problem (i.e., the age variable being correlated with other firm characteristics) as pointed out by Aga and Francis [32]. The effect of export on firm exit is negative but not statistically significant. In the same vein, the results show that the presence of foreign capital ownership does not improve firm survival nor does firm productivity matter for better survival prospects.

The results also provide evidence for the effect of having at least one female as part of the business owners. The effect is negative and statistically significant with an estimated exit probability that declines by roughly 4 percentage points. Thus, this contradicts the popular belief that female-owned businesses are more vulnerable than their counterpart. Similarly, there is significant evidence that firms with a history of purchasing fixed assets using bank loans are less likely to exit the market. The risk of exiting the markets drops by 12 percentage points for asset-purchasing firms relative to their counterpart. This implies that improving private sector access to financial credits for investment can help improve business success. In line with evidence in the literature (e.g., [32]), micro-, small, and medium enterprises (MSMEs) face a greater risk of exit (estimated at around 8 percentage points) compared to their opponent (large firms). The finding is not surprising given the fact that MSMEs are more constrained in several areas and thus more vulnerable to business failure.

## Conclusions

This paper set out to investigate how business climate accounts for firm exit in Nigeria using firm-level data from the World Bank Enterprise Survey. Despite being the largest economy in Africa, Nigeria’s business environment is faced with diverse challenges that impede business success. The state of the business climate in Nigeria is such that is characterized by several obstacles such as insecurity, infrastructural deficit, poor institutional quality (most especially corruption), unfavorable tax policies, and excessive regulations from the government. Business operation in the country is hindered by a number of these constraints, with electricity access deficit as the most binding constraint, and has over the years remained the most cited obstacle to the smooth flow of business activities in Nigeria. Despite the privatization of the sector in the recent past, the sector is still faced with a huge disparity between generation capacity and demand which resulted to most businesses using costly standby diesel generators to meet their energy demands. According to the 2014 Enterprise Survey, over 70% of interviewed businesses in Nigeria owned standby diesel generators which account for the bulk of their energy use. Likewise, the country’s transport sector (most especially road transport) is faced with poor funding and neglect by the government, with consequences such as disruption in the commodity supply chain and increase in business operational costs. Additional constraints in the business environment include unfavorable tax policies from the government, excessive regulations, and a poor institutional environment.

This study took a holistic view of the business environment by aggregating 15 firm-level indicators into a composite index and estimates the effect on the firm likelihood of exit using a binary probit model. The analysis suggests that the poor state of the business climate in Nigeria increases firms' risk of exit from the market, with an additional level of constraints implying that more firms will significantly exit the market. The practical implication of this finding infers that policymakers should gear more efforts toward improving the condition of the business environment in Nigeria through carefully designed policies that can foster private sector development. Such policies should among others increase government investment in critical infrastructure, eliminate destructive tax policies, and maintain a healthy macroeconomic environment, which by implication, go a long way to improve business longevity and contributes to national development.

## Data Availability

Data used for this study is derived from a source in the public domain—World Bank Enterprise Survey and available for registered users *at* https://login.enterprisesurveys.org/content/sites/financeandprivatesector/en/library/library-detail.html/content/dam

## Footnotes

1 The results of the Correlation Analysis are available from the author upon request.
